# Association of osteotomy, age, and component fixation with the outcomes of total hip arthroplasty in patients with hip dysplasia: a Dutch population-based registry study

**DOI:** 10.2340/17453674.2024.41383

**Published:** 2024-09-13

**Authors:** Milou F T HÜSKEN, Joëll MAGRÉ, Koen WILLEMSEN, Liza N VAN STEENBERGEN, Mirthe H W VAN VEGHEL, Harrie WEINANS, Ralph J B SAKKERS, Joris E J BEKKERS, Bart C H VAN DER WAL

**Affiliations:** 1 Clinical Orthopedic Research Center–mN, Diakonessenhuis, Zeist; 2 Department of Orthopedic Surgery, Diakonessenhuis, Utrecht/Zeist; 3 Department of Orthopedic Surgery, University Medical Center Utrecht, Utrecht; 4 3D Lab, Division of Surgical Specialties, University Medical Center Utrecht, Utrecht; 5 Dutch Arthroplasty Register (LROI), ’s Hertogenbosch; 6 Department of Orthopedics, Radboud University Medical Center, Nijmegen; 7 Department Biomechanical Engineering, Delft University of Technology, Delft, The Netherlands

## Abstract

**Background and purpose:**

Hip dysplasia can present challenges for total hip arthroplasty (THA) due to anatomic abnormalities. We aimed to assess the association of age, sex, osteotomies prior to THA, and fixation method on 5- and 10-year revision-free implant survival and patient-reported outcome measures (PROMs) of THAs in patients with hip dysplasia.

**Methods:**

Using Dutch Arthroplasty Register data, we studied hip dysplasia patients receiving primary THAs in 2007–2021 (n = 7,465). THAs were categorized by age, pelvic osteotomy prior to THA (yes/no), and fixation (cemented, uncemented, hybrid, reverse hybrid). Kaplan–Meier and multivariable Cox models were used to determine 5- and 10-year revision-free implant survival and adjusted hazard ratios including 95% confidence intervals (CIs). Reasons for revision and PROMs were compared within the categories.

**Results:**

We found a 10-year revision-free implant survival of 94.9% (CI 94.3–95.5). Patients younger than 50 years had a 10-year implant survival of 93.3% (CI 91.9–94.7), Patients with prior pelvic osteotomy had a 10-year implant survival of 92.0% (CI 89.8–94.2). Fixation method and sex were not associated with implant survival. Patients with a prior pelvic osteotomy had more revisions due to cup loosening and reported lower PROM scores than patients without earlier osteotomy.

**Conclusion:**

5- and 10-year revision-free implant survival rates of THA for hip dysplasia are 96.4% and 94.9%. Age and prior osteotomies were associated with decreased implant survival rates in patients with hip dysplasia, while fixation method was not. Prior osteotomies were also associated with reduced PROM scores.

Roughly 10% of hip osteoarthritis (OA) patients exhibit underlying hip dysplasia [1,2]. The hip anatomy in dysplastic hips with shallow, anterolaterally deficient, and anteverted acetabula providing inadequate femoral head coverage can pose challenges when performing a total hip arthroplasty (THA). Surgeons may perform acetabular reconstructions or femoral osteotomies to address bone deficiency and restore the anatomical center of rotation. This led historically to poorer implant survival rates in childhood hip disorders, with an increased risk of revision in the first 6 months postoperatively [3-5].

Besides altered anatomy, a younger age at the time of THA, and the type of implant prior hip joint osteotomies seem to contribute to lower implant survival rates in hip dysplasia patients. Younger patients generally experience lower THA implant survival rates, a trend that is also evident in those with hip dysplasia [6,7]. According to Furnes et al., and Engesæter et al. such lowered implant survival rates in patients with dysplasia of the hip are due to the use of inferior uncemented implants [8,9]. In addition, although osteotomies performed around the hip joint before THA did not seem to significantly impact implant survival rates previously [10], they were associated with slightly lower functional outcomes and higher complication rates [11].

Existing data on THA survival rates in hip dysplasia patients often have a selection bias as it is often derived from subpopulations with unique implants, fixation techniques, or age groups that limit generalizability. Moreover, previous registry studies report no patient-reported outcome measures (PROMs) or possible association with prior osteotomy. Therefore, we aimed to examine the association of age, sex, osteotomy prior to THA, and fixation method on 5- and 10-year revision-free implant survival, reasons for revision, and PROMs of THAs in patients with hip dysplasia.

## Methods

This population-based study utilized data from the Dutch Arthroplasty Register (LROI), a national registry that has been collecting data on orthopedic interventions since 2007. The registry has achieved over 95% coverage of Dutch hospitals since 2012 [12], with a current completeness of 99% in primary hip arthroplasties and 97% of revision THAs. The STROBE guidelines were adhered to for reporting the study results.

### Data source

The LROI contains patient, procedure, and prosthesis characteristics as well as validated PROMs: EuroQol 5D (EQ-5D), Oxford Hip Score (OHS), and Hip Disability and Osteoarthritis Outcome Score with Physical Function Short Form (HOOS-PS). While patient characteristics have been recorded since the inception of the LROI, body mass index (BMI) and PROMs have been collected from 2014 onward. The LROI does not collect data on the degree of dysplasia (Crowe grade) or the type of osteotomy performed (e.g., Chiari osteotomy, peri-acetabular osteotomy, Ganz osteotomy). For each component of the THA, a product number is registered to identify the prosthesis characteristics. The patient’s status is obtained on a regular basis from the national insurance database, which records all deaths of Dutch citizens. The loss to follow-up due to emigration is unknown but is expected to be limited.

### Patients and parameters

This study included all THAs with a registered diagnosis of hip dysplasia in the LROI between 2007 and 2021. The diagnosis of hip dysplasia is based on the clinicians’ view, using radiographs without validation by a second interpreter. Patient, procedure, and prothesis characteristics included in this study were age at the time of the procedure, BMI, sex, American Society of Anesthesiologists (ASA) class, fixation method (cemented, uncemented, hybrid [cemented femur], or reverse hybrid [cemented cup]), and pelvic osteotomy prior to THA (yes or no).

### Outcome

Outcome measures included the revision-free implant survival of THA at 5 and 10 years, stratified by age group (< 50 and ≥ 50 years), prior pelvic osteotomy, and fixation method. Implant survival was also analyzed across 10-year age ranges: < 30, 30–39, 40–49, 50–59, 60–69, 70–79, and 80 years and older. Survival time was calculated from the date of primary THA to the first revision arthroplasty for any reason, death of the patient, or the end of the study follow-up on January 1, 2023. For functional outcomes, only patients with both preoperative and 12-month postoperative PROMs were included. Due to insufficient data, fixation method comparisons were not feasible

### Statistics

To compare baseline data and reasons for revision, the properties and distribution of the variables were checked for appropriate use of parametric or non-parametric tests, and were described using either median and range, mean with standard deviation (SD) or percentages. The independent samples t-test or Mann–Whitney U test were used to compare continuous variables between groups, and the chi-square test was used for categorical variables. Reasons for revision were described according to subgroup and compared using a chi-square. Missing data was assumed to be missing at random; therefore, we did not perform any imputation and analyzed only the available data.

Kaplan–Meier survival analyses were performed to determine the 5- and 10-year revision-free implant survival rates for THA in patients with hip dysplasia, stratified by age, prior pelvic osteotomy, and fixation group. Multivariable Cox proportional hazard ratios (HR) were performed to compare adjusted revision rates between age groups, osteotomy, and fixation method groups of THAs. Adjustments were made for age at surgery, sex, osteotomy, and fixation method to discriminate independent risk factors for revision arthroplasty. For all covariates added to the model, we inspected the log-minus-log curves. The proportional hazards assumption was met.

To assess functional outcomes, propensity score matching using the nearest neighbor method accounted for differences in patient population between < 50 and ≥ 50 years age groups, prior pelvic osteotomy, and non-osteotomy groups. Groups were matched 1:1 on sex, ASA class, BMI, age, or prior pelvic osteotomy. A caliper width of 0.05 was used. All standardized mean differences were < 0.100 post-matching. The baseline and 12-month postoperative PROMs, as well as the PROM difference (improvement) between those moments, were compared using Mann–Whitney U tests for each questionnaire.

Statistical analyses were performed using SPSS (IBM Corp, Armonk, NY, USA). P < 0.05 was considered statistically significant. For the 95% confidence intervals (CI), we assumed that the number of observed cases followed a Poisson distribution. R was used for matching the data (R Foundation for Statistical Computing, Vienna, Austria), and Prism (https://www.graphpad.com/) for producing graphs.

### Ethics, registration, data sharing, funding, use of AI, and disclosures

This study did not fall under the scope of the Medical Research Involving Human Subjects Act (WMO) in the Netherlands. Consequently, ethical approval was only required and obtained from the local ethics committee of the LROI (Landelijke Registratie Orthopedische Implantaten), which is the registry used in this study. The study was registered in the LROI, and all procedures were conducted in accordance with the guidelines provided by this registry. The data used in this research is available upon reasonable request, subject to the approval of the LROI. There was no use of Artificial Intelligence (AI) in the analysis or any part of this study. This research received no specific grant from any funding agency in the public, commercial, or not-for-profit sectors.

HW has received various research grants and contracts, holds minority shares in Replasia BV, Uplanner BV, and Amotio BV, and has patents pending in related areas. RS is listed as an inventor on several patents related to orthopedic implants and holds minority shares in Uplanner BV and Replasia BV. He also serves in leadership roles in the European Paediatric Orthopaedic Society and the Osteogenesis Imperfecta Federation Europe, among other boards. BvdW holds patents related to orthopedic implants, has minority shares in Replasia BV, Amotio BV, and Uplanner BV, and is a member of advisory boards for these companies. The other authors declare no competing interests. Complete disclosure of interest forms according to ICMJE are available on the article page, doi: 10.2340/17453674.2024.41383

## Results

**Figure 1:**
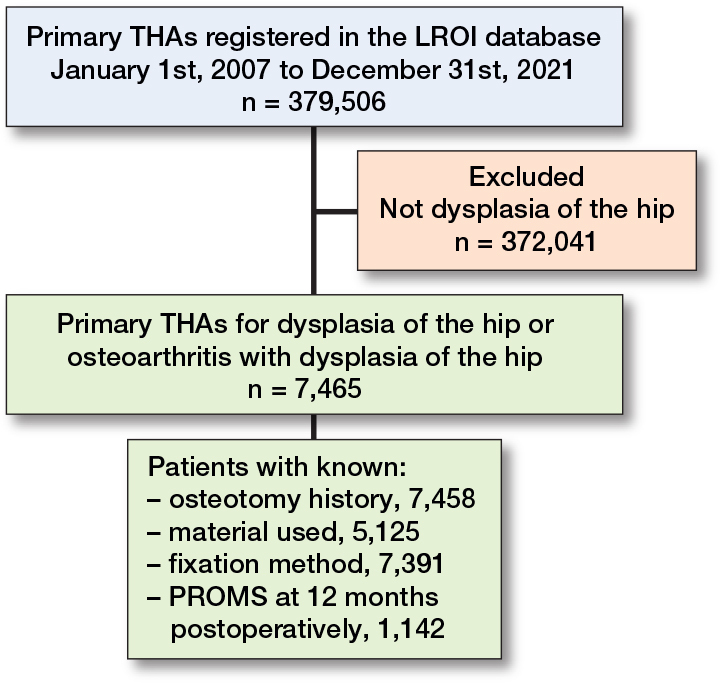
Patient flow of study population. THA = total hip arthroplasty, LROI = Dutch Arthroplasty Register, PROMs = patient-reported outcome measures.

7,465 primary THAs with a hip dysplasia diagnosis were registered in the LROI between 2007 and 2021 (Figure 1). Among these, 2,377 THAs (32%) were performed in patients aged under 50 years and 1,124 THA procedures (15%) reported a prior osteotomy. Notably, 71% of the study population received cementless THAs and 92% of all procedures were performed in patients classified as ASA I–II (Table 1). The most frequently registered reason for revision was dislocation (28% of all revisions).

**Table 1:** Patient and procedure characteristics of the complete study population (N = 7,465). Values are count (%) unless otherwise specified

Patient characteristics	
Age at surgery, mean (SD)	56 (14)
Female	5,209 (70)
ASA class	
I	2,994 (40)
II	3,823 (52)
III–IV	565 (7.7)
Prior pelvic osteotomy	1,124 (15)
BMI **^a^**, median (range)	26.0 (10.4–56.8)
Smoking **^a^**	602 (8.1)
Procedure characteristics	
Dual mobility cup	293 (4.0)
Fixation	
Cemented	1,222 (17)
Cementless	5,241 (71)
Hybrid	236 (3.2)
Reverse hybrid	699 (9.4)

SD = standard deviation, ASA = American Society of Anesthesiologists score, BMI = body mass index.

a Since 2014 registered in the LROI.

### Patient characteristics

THAs performed in patients aged under 50 years had a higher incidence of prior osteotomies (30% vs 8%, difference: 22% [CI 19.7–23.7]) than patients 50 years and older. The THAs were more frequently performed in females (75% vs 67% for ≥ 50-year-old patients, 8% more [CI 5.8–10.1]). Moreover, for THAs in patients under 50 years, a reverse hybrid fixation was used more often (12% vs 8%, 4% more [CI 2.6–5.6] in young patients) (Table 2). No differences were seen in reasons for revision between the 2 age groups (Table 3),

**Table 2a:** Comparison of patient and surgery characteristics for THA patients with hip dysplasia according to age, and prior pelvic osteotomy. Values are count (%) unless otherwise specified. A difference of < 0.5% is noted as 0

	Age groups		Difference % points (CI)	P	Osteotomy		Difference % points (CI)	P
	< 50 years n = 2,377	> 50 years n = 5,081			Prior pelvic n = 1,124	No n = 6,334		
Mean age (SD)	40 (8)	63 (9)			45 (14)	58 (13)		
< 50 years	–	–			710 (63)	1,667 (26)	37 (34–40)	**^a^**
Median BMI	26.0	26.7			25.4	26.8		
min.	10.4	14.9			14.7	10.4		
max.	50.0	56.8			43.0	56.8		
BMI < 25	(40)	(39)	1.2 (–1.8 to 4.2)		(45)	(38)	6.7 (2.7 to 11)	**^a^**
Female	1,788 (75)	3,416 (67)	8.0 (5.8 to 10)	**^a^**	893 (80)	4,316 (68)	12 (8.9 to 14)	**^a^**
Fixation								
Cemented	370 (16)	852 (17)	–1.2 (–3.0 to 0.6)		258 (23)	964 (15)	7.8 (5.3 to 11)	**^a^**
Cementless	1,632 (69)	3,602 (71)	–2.2 (–4.2 to 0.2)		637 (57)	4,604 (73)	–16 (–18.8 to –13)	**^a^**
Hybrid	60 (2.5)	176 (3.5)	–1.0 (–1.8 to 0.1)		40 (3.6)	196 (3.1)	0.5 (–0.6 to 1.6)	
Reverse hybrid	288 (12)	411 (8.1)	4.0 (2.6 to 5.6)	**^a^**	173 (15)	526 (8.3)	7.1 (5.0 to 9.5)	**^a^**
Unknown	27 (1.1)	40 (0.8)	0		16 (1.4)	51 (0.8)	0	
Inlay								
Ceramics	33 (1.4)	19 (0.4)	1.0 (0.5 to 1.5)	**^a^**	12 (1.1)	40 (0.6)	0	
Oxidized zirconium	183 (7.7)	405 (8.0)	0		46 (4.1)	543 (8.6)	–4.5 (–5.8 to –3.1)	**^a^**
Cobalt chrome	36 (1.5)	83 (1.6)	0		12 (1.1)	107 (1.7)	–0.6 (–1.3 to 0.1)	
XL PE	1,003 (42)	2,178 (43)	–0.7 (–3.1 to 1.7)		417 (37)	2,766 (44)	–6.5 (–9.5 to –3.4)	**^a^**
XL PE + AO	176 (7.4)	350 (6.9)	0		68 (6.0)	459 (7.2)	–1.2 (–2.7 to 0.3)	
Std PE	157 (6.6)	497 (9.8)	–2.8 (–4.1 to –1.5)	**^a^**	95 (8.5)	560 (8.8)	0	
Unknown	789 (33)	1,649 (30)	2.7 (0.7 to 5.3)	**^a^**	474 (42)	1,866 (29)	13 (9.3 to 16)	**^a^**
Prior osteotomy	710 (30)	414 (8.1)	22 (20 to 24)	**^a^**				

BMI = body mass index, XL PE = cross-linked polyethylene, AO = antioxidant, Std PE = standard polyethylene.

a Significant difference.

**Table 2b:** Comparison of patient and surgery characteristics for THA patients with hip dysplasia according to fixation method. Values are count (%) unless otherwise specified

	Component fixation				P
	Cementless n = 5,234	Cemented n = 1,222	Rev. hybrid n = 699	Hybrid n = 236	
Mean age (SD)	56 (13)	59 (18)	52 (13)	60 (16)	**^a^**
< 50 years	(31)	(30)	(41)	(25)	
Median BMI	26.0	26.0	26.2	25.9	**^a^**
min.	10.4	14.7	15.6	18.0	
max.	56.8	56.5	51.7	37.8	
BMI < 25	(39)	(40)	(38)	(44)	
Female	3,538 (68)	927 (76)	524 (75)	174 (74)	
Inlay					
Ceramics	45 (0.9)	0 (0)	0 (0)	7 (3)	**^a^**
Oxidized zirconium	545 (10)	4 (0.3)	0 (0)	38 (16)	
Cobalt chrome	114 (2.2)	1 (0.1)	0 (0)	4 (1.7)	
XL PE	3,009 (57)	23 (1.9)	17 (2.4)	117 (50)	
XL PE + AO	415 (7.9)	48 (3.9)	39 (5.6)	19 (8.1)	
Std PE	511 (10)	55 (4.5)	58 (8.3)	22 (9.3)	
Unknown	602 (12)	1,091 (89)	585 (84)	29 (12)	
Prior osteotomy	637 (12)	258 (21)	173 (25)	40 (17)	

Rev. = Reverse; also see Table 2a for abbreviations.

**Table 3a:** Comparison of reasons for revisions for THA patients with hip dysplasia according to age, and prior pelvic osteotomy. Values are count (%) unless otherwise specified. A difference of < 0.5% is noted as 0

	Age groups		Difference % points (CI)	P	Osteotomy		Difference % points (CI)	P
	< 50 years n = 2,377	> 50 years n = 5,081			Prior pelvic n = 1,124	No n = 6,334		
Revised	119	178			66	233		
Follow-up years, mean (SD)	6.2 (3.8)	6.8 (3.8)			6.6 (3.8)	6.4 (4.0)		
range	0–15	0–15			0–15	0–15		
Reason for revision **^a^**								
Dislocation	30 (25)	53 (30)	–4.5 (–15 to 5.7)		16 (24)	67 (29)	–4.6 (–16 to 7.3)	
Infection	16 (13)	27 (15)	–1.8 (–9.8 to 6.4)		11 (17)	32 (14)	3.0 (–7.1 to 13)	
Loosening femur	14 (13)	35 (20)	–7.9 (–16 to 0.3)		7 (11)	42 (18)	–7.4 (–16 to 1.5)	
Loosening acetabulum	22(18)	26 (15)	2.9 (–4.8 to 13)		18 (27)	30 (13)	14 (2.8 to 26)	**^b^**
Periarticular ossifications	2 (1.7)	6 (3.4)	–1.7 (–5.2 to 1.8)		4 (6.1)	4 (2.7)	3.4 (–1.6 to 10)	
Periprosthetic fracture	10 (8.4)	20 (11)	–2.8 (–9.6 to 4.0)		3 (4.5)	27 (12)	–7.1 (–14 to –0.6)	**^b^**
Wear	9 (7.6)	6 (3.4)	4.2 (–1.2 to 9.6)		4 (6.1)	11 (4.7)	1.4 (–5.0 to 7.7)	
Other	42 (35)	37 (21)	14 (4.1 to 25)	**^b^**	15 (23)	65 (28)	–5.2 (–17 to 6.5)	

a Multiple reasons could be marked to be the reason for revision; therefore, the percentages add up to more than 100.

b Significant difference.

**Table 3b:** Comparison of reasons for revisions for THA patients with hip dysplasia according to fixation method. Values are count (%) unless otherwise specified

	Component fixation				P
	Cementless n = 5,234	Cemented n = 1,222	Rev. hybrid n = 699	Hybrid n = 236	
Revised	219	39	25	6	
Follow-up years, mean (SD)	6.7 (3.8)	6.4 (3.8)	6.2 (3.8)	5.9 (3.7)	
range	0–15	0–15	0–15	0–14	
Reason for revision **^a^**					
Dislocation	58 (26)	11 (28)	7 (28)	3	
Infection	35 (16)	3 (7.7)	3 (12)	0	
Loosening femur	38 (17)	8 (21)	2 (8.0)	0	
Loosening acetabulum	25 (11)	10 (26)	9 (36)	1	**^b^**
Periarticular ossifications	5 (2.3)	1 (2.6)	2 (8.0)	0	
Periprosthetic fracture	21 (10)	5 (13)	2 (8.0)	0	
Wear	10 (4.6)	1 (2.6)	3 (12)	0	
Other	69 (29)	4 (10)	5 (20)	2	

a, b See Table 3a. Rev. = Reverse.

THAs following a prior pelvic osteotomy were performed at a younger age than in patients without a prior pelvic osteotomy (45 years [SD 14] vs 58 years [SD 13]). THAs following a prior pelvic osteotomy were more often performed in females (80% vs 68%, 12% more [CI 8.9–14.1]) and surgeons used full cemented or reverse hybrid fixation more often (respectively 8% and 7% more) for those with than those without a prior pelvic osteotomy (Table 2). However, THAs for patients with a prior pelvic osteotomy were still revised more often due to loosening of the acetabular cup than for patients without prior osteotomy (27% vs 13%, 14% more [CI 2.8–26.0]), but less due to periprosthetic fractures (5% vs 12%, 7% less [CI 0.6–13.5] than for those without a prior pelvic osteotomy (Table 3).

THAs in patients with hybrid fixation had the highest mean age (60 years vs 52–59 years in the other groups), and in line with the results mentioned above, the reverse hybrid fixation had the highest proportion with prior osteotomies (25% vs 12–21% prior osteotomies in the other fixation methods) (Table 2). No specific reasons for revision could be linked to a fixation method (Table 3).

### Implant survival

**Figure 2:**
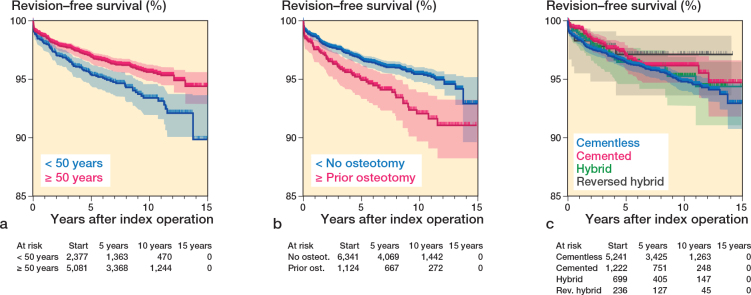
Kaplan–Meier revision-free survival rates according to (a) age (< 50 years, ≥ 50 years; P < 0.01), (b) pelvic osteotomy before THA (P < 0.01), (c) fixation method (P = 0.4).

The revision-free implant survival of THAs in patients with dysplasia of the hip was 96.4% (CI 96.0–96.8) at 5 years and 94.9% (CI 94.3–95.5) at 10 years. THAs performed in patients under 50 years had a lower implant survival: 95.3% (CI 94.3–96.3) at 5 years and 93.3% (CI 91.9–94.7) at 10 years, significantly differing from those 50 years and older with 96.9% (CI 96.3–97.5) at 5 years and 95.6% (CI 94.8–96.4) at 10 years (Figure 2). Every 10-year increase in age resulted in a higher revision-free implant survival (Table 4). The multivariable Cox regression analysis showed an HR for revision of 1.3 (CI 1.0–1.7) for THAs in patients < 50 years versus those aged ≥ 50 years, adjusted for sex, osteotomy, and fixation method (Table 5).

**Table 4:** 5- and 10-year revision free survival of THA stratified by age group. Values are percentages (%)

Age group	n	Revision-free survival (CI)	
		5 years	10 years
< 30	303	92.4 (88.9–95.9)	90.4 (85.9–94.9)
30–39	560	95.7 (93.9–97.5)	92.4 (89.3–95.5)
40–49	1,514	95.7 (94.5–96.9)	94.2 (92.6–95.8)
50–59	2,027	95.9 (94.9–96.9)	94.1 (92.7–95.5)
60–69	1,726	97.2 (96.4–98.0)	95.9 (94.7–97.1)
70–79	1,055	97.7 (96.7–98.7)	97.3 (96.1–98.5)
≥ 80	273	99.2 (98.0–100)	98.6 (95.5–100)

CI = 95% confidence intervals.

**Table 5:** Multivariable Cox regression with hazard ratio for revision adjusted for age, previous pelvic osteotomies, and fixation method

Covariate	HR (CI)
Age < 50 years	1.3 (1.0–1.7) **^a^**
Female sex	0.9 (0.7–1.2)
Prior pelvic osteotomy	1.5 (1.1–2.0) **^a^**
Fixation	
Cemented	1 (Ref.)
Cementless	1.3 (0.9–1.9)
Hybrid	0.8 (0.4–2.1)
Reverse hybrid	1.1 (0.7–1.8)

HR = hazard ratio, CI = 95% confidence interval.

a Significant difference.

THAs in patients with a prior pelvic osteotomy had a significantly lower revision-free implant survival of 94.8% (CI 93.4–96.2) at 5 years and 92.0% (CI 89.8–94.2) at 10 years, compared with patients without a prior pelvic osteotomy with 96.7% (CI 96.3–97.1) at 5 years and 95.4% (CI 94.8–96.0) at 10 years (Figure 2). Adjusted analyses showed an HR for revision of 1.5 (CI 1.1–2.0) for THAs in patients with a prior pelvic osteotomy compared with patients without osteotomy, adjusted for age, sex, and fixation method (Table 5).

The different fixation methods were not significantly associated with changes in implant survival (Figure 2); cemented, hybrid, and reverse hybrid compared with cementless resulted in an adjusted HR of respectively 0.9 (CI 0.5–1.1), 0.7 (CI 0.3–1.5), and 0.8 (CI 0.6–1.3) (Table 5).

### Patient-reported outcome measures

Patients younger than 50 years had lower preoperative PROMs compared with older patients in 2 out of 3 questionnaires. Specifically, younger patients scored a median of 0.48 points for the EQ-5D, while older patients scored a median of 0.60 points (scale 0–1, 1 being an optimal score). Similarly, mean preoperative OHS was lower, with 22 points versus 25 in older patients (scale 0–48, 48 being an optimal score), indicating that young patients experience more limitations in daily living and more symptoms preoperatively. However, the 12-month postoperative scores were comparable across all age groups, with a greater improvement for THA patients younger than 50 years with the same questionnaires; EQ-5D: 0.34 vs 0.28 points improvement, OHS: 21.5 vs 19 points improvement for 12 months (Table 6). Although the HOOS-PS scores were slightly better in the older age group (51 vs 46 on a scale of 100–0, with 0 being an optimal score), the difference was not statistically significant between the age groups (Table 6).

**Table 6:** Comparison of patient reported outcome measures (PROMs) according to age groups. Values are median (range)

PROM	Age groups – Matched population		P
	< 50 years n = 318	≥ 50 years n = 318	
EQ-5D			
Preoperative	0.49 (–0.35 to 0.95)	0.60 (–0.10 to 0.95)	**^a^**
12 months p.o.	0.89 (0.20 to 1.00)	0.95 (0.15 to 1.00)	
Delta	0.34 (–0.24 to 0.97)	0.28 (–0.36 to 0.88)	
OHS			
Preoperative	22 (3 to 48)	25 (7 to 47)	**^a^**
12 months p.o.	45 (12 to 48)	45 (1 to 48)	
Delta	21.5 (–10 to 45)	19 (–23 to 40)	**^a^**
HOOS-PS			
Preoperative	51 (100 to 0)	46 (100 to 9)	
12 months p.o.	9 (91 to 0)	9 (68 to 0)	
Delta	38 (–29 to 100)	37 (–29 to 82)	

p.o. = postoperative. EQ-5D = EuroQol 5D, OHS = Oxford Hip Score, HOOS-PS = Hip disability and Osteoarthritis Outcome Score.

a Significant difference.

EQ-5D showed no differences in index scores based on prior pelvic osteotomy. However, patients with osteotomy demonstrated slightly poorer 12-month postoperative scores on the OHS, with a median score of 44 vs 46 in non-osteotomy patients. They also showed less improvement on the HOOS-PS, with 33 vs 38 points improvement over 12 months, indicating a negative impact of a prior pelvic osteotomy on THA outcomes (Table 7).

**Table 7:** Comparison of patient-reported outcome measures (PROMs) according to prior pelvic osteotomy. Values are median (range)

PROM	Pelvic osteotomy – Matched population		P
	Prior pelvic n = 155	No n = 155	
EQ-5D			
Preoperative	0.55 (–0.10 to 0.84)	0.55 (–0.08 to 0.95)	
12 months p.o.	0.82 (0.25 to 1.00)	0.88 (0.15 to 1.00)	
Delta	0.28 (–0.08 to 0.94)	0.33 (–0.22 to 0.97)	
OHS			
Preoperative	23 (5 to 44)	23 (3 to 48)	
12 months p.o.	44 (15 to 48)	46 (18 to 48)	**^a^**
Delta	21 (–9 to 39)	21 (0 to 45)	
HOOS-PS			
Preoperative	48.5 (100 to 0)	51 (100 to 5)	
12 months p.o.	11 (68 to 0)	9 (91 to 0)	
Delta	33 (–15 to 100)	38 (–29 to 95)	**^a^**

For abbreviations, see Table 6.

## Discussion

We aimed to assess the association of age, sex, osteotomies prior to THA, and fixation method with the 5- and 10-year revision-free implant survival and PROMs of THAs in patients with hip dysplasia. We found a lower revision-free rate in patient with a younger age and those with a history of prior osteotomies. THA patients with a prior osteotomy were associated with lower PROMs. We found no association between fixation method or sex on the implant survival in hip dysplasia patients.

The 10-year revision-free implant survival rate of THA in this hip dysplasia population was 94.9% (CI 94.3–95.5), which is comparable to the LROI data on THAs for all diagnoses with a 10-year implant survival rate of 95.3% (CI 95.2–95.4). The current study population was younger compared with the LROI data on “THAs for all diagnoses,” aged on average 56 years (SD 14) vs 69 years (SD 11) respectively [13]. These rates are also comparable with a 10-year implant survival of 95.6% (CI 94.8–96.4) in the more comparable group of hip dysplasia patients aged 50 years and older with a mean age of 63 years (SD 9) in our study. The comparable revision rates between THAs for hip dysplasia and THAs for other indications are in accordance with other registry studies [5,8,14,15].

### Association of age with survival and functional outcomes

Revision-free implant survival rates were negatively associated with patient age. Young (< 50 years) hip dysplasia patients had a lower 5-year and 10-year implant survival (95.3% and 93.3%, respectively). These rates are comparable with findings by Kuijpers et al. [16] for young Dutch patients receiving a THA for all diagnoses. They described 5-year implant survival rates between 93.6% and 96.1% for each year from 2007–2011. International studies also report 10-year implant survival rates comparable or lower than our results for patients < 50 years. For example Mei et al. [7] reported an implant survival rate of 94.6% after 10 years in patients younger than 55 years, all in non-dysplastic populations. Thus, age has a stronger association with the 10-year revision-free implant survival than the presence of hip dysplasia, which is supported by another study from the LROI: “Pediatric hip disorders are not associated with an increased 10-year revision risk after total hip arthroplasty under the age of 55: results from the Dutch Arthroplasty Registry”[17]. Since the LROI started in 2007, there is limited data available to calculate an implant survival rate beyond 10 years. Our study also showed that younger patients (< 50 years) with hip dysplasia have worse preoperative PROMs than the older patients, and have a bigger improvement with similar outcomes after surgery. The differences, however, are small and not clinically relevant based on the minimal clinical important difference (MCID): > 0.03–0.52 on the EQ-5D [18], ≥ 5 on the OHS [19], and ≥ 6 on the HOOS-PS [20].

### Association of prior osteotomies with survival and functional outcomes

Prior pelvic osteotomy significantly reduced the 10-year revision-free implant survival rates of THA to 92.0% compared with patients without osteotomy with 95.4%. Multiple studies reported higher intraoperative blood loss, lower consistency in cup positioning, and possibly compromised patient-reported outcomes [11]; however, a reduction in implant survival in patients with a pelvic osteotomy before THA was not reported before. The association with prior osteotomies has mostly been researched in smaller populations, stating no difference in THA survival [10]. Our study found a higher incidence of cup loosening in patients who had undergone prior osteotomies. This could be attributed to the challenges in achieving optimal positioning of the acetabular component, which is crucial in establishing the new hip center of rotation and directly influences hip biomechanics and wear rates. A possible explanation for the higher rates of loosening could be that these patients had more severe dysplasia (needing an osteotomy at an early age), which may have made it more challenging to achieve optimal acetabular cup positioning. Severity of dysplasia, however, was not registered in the LROI. Studies with patient-specific autografts [21] or custom-made devices [22] have demonstrated 10-year implant survival rates of 94% and 95.4% respectively, comparable to our population without prior pelvic osteotomies. This indicates that THA could be an effective treatment option for all grades of hip dysplasia, if customized to the specific needs of a patient.

According to Migaud et al. [23], the type of prior pelvic osteotomy did not affect the outcomes; they published a 15-year implant survival rate of 87% independent of the type of prior pelvic osteotomy (i.e., shelf arthroplasty/Chiari osteotomy/femoral osteotomy/Milch osteotomy). The severity of hip dysplasia, however, will likely be associated with the outcomes. Unfortunately, the LROI data does not contains specifics on prior hip surgery type. While lower PROMs were associated with osteotomy prior to THA in line with existing literature, these differences are again too small to be clinically relevant when considering MCIDs.

### Association of fixation with survival and functional outcomes

It has previously been described that poorer outcomes in cases with dysplasia are mostly due to the use of inferior uncemented implants [8,9]. However, in our study the different fixation methods were not significantly associated with changes in revision-free implant survival. This is in accordance with previous reviews and registry studies analyzing the use of cement for implants [24,25]. Even after adjustment for age and prior osteotomies, the implant survival rates still did not differ between the fixation methods. Matching of these groups for an analysis of the PROMs was unfortunately not possible, due to the low numbers.

### Limitations and strengths

The degree of dysplasia and the type of osteotomy were not registered, and femoral osteotomies were not registered as such. Additionally, there is a potential for misclassification as less than 2% of the total number of primary THAs had been diagnosed with hip dysplasia, which is considerably lower than the 4–13% attribution of hip dysplasia to osteoarthritis claimed in the literature [1,2]. However, with more than 95% completeness of registration of arthroplasties in Dutch hospitals since 2012 [12], the LROI database should contain nearly all patients with dysplasia and a THA for the past 14 years in The Netherlands. Cases of very mild hip dysplasia (Crowe 1) may have been overlooked compared with more severe forms, due to the single observation by the treating surgeon. Under-registration in milder or non-symptomatic cases might bias this study data, possibly reflecting more severe hip dysplasia cases.

Although the 10-year revision-free implant survival rates of THA in patients with hip dysplasia are excellent, this study highlights the significant association of patient age and prior osteotomies with implant survival and functional outcomes. Particularly for patients with severe hip dysplasia, who are often young when they develop symptoms, the data underscore the importance of personalized treatment strategies.

### Conclusion

5- and 10-year revision-free implant survival rates of THA for hip dysplasia are 96.4% and 94.9%. Age and prior osteotomies were associated with decreased implant survival rates, while fixation method is not. Prior osteotomies were also associated with reduced PROMs.

## Notes

The study was conceptualized and the methodology was developed by MH, JM, LvS, MV, HW and BvdW. Data collection was conducted by the Dutch Arthroplasty Register (LROI), with LvS overseeing the data monitoring process. MH and MV performed the formal analysis of the data. The original draft of the manuscript was written by MH, and the manuscript was reviewed and edited by all authors. MH was responsible for the visualization, including the creation of figures and charts. Supervision of the project was provided by JB, RS, HW and BvdW. All authors approved the final version of the manuscript.

Handling co-editors: Marianne Westberg and Robin Christensen

*Acta* thanks Ove Nord Furnes and Lars Nordsletten for help with peer review of this manuscript.
